# Microscale Mapping of Structure and Stress in Barium Titanate

**DOI:** 10.6028/jres.125.013

**Published:** 2020-04-04

**Authors:** Jane A. Howell, Mark D. Vaudin, Lawrence H. Friedman, Robert F. Cook

**Affiliations:** 1 National Institute of Standards and Technology, Gaithersburg, MD 20899, USA

**Keywords:** barium titanate (BaTiO_3_), domain, electron backscatter diffraction, microdomain, single crystal, strain, stress

## Abstract

Cross-correlation of electron backscatter diffraction (EBSD) patterns was used to generate rotation, strain, and stress maps of single-crystal tetragonal barium titanate (BaTiO_3_) containing isolated, small, sub-micrometer *a* domains separated from a *c*-domain matrix by 90° domain boundaries. Spatial resolution of about 30 nm was demonstrated over 5 μm maps, with rotation and strain resolutions of approximately 10^−4^. The magnitudes of surface strains and, especially, rotations peaked within and adjacent to isolated domains at values of approximately 10^−2^, *i.e.*, the tetragonal distortion of BaTiO_3_. The conjugate stresses between *a* domains peaked at about 1 GPa, and principal stress analysis suggested that stable microcrack formation in the *c* domain was possible. The results clearly demonstrate the applicability of EBSD to advanced multilayer ceramic capacitor reliability and strongly support the coupling between the electrical performance and underlying mechanical state of BaTiO_3_-containing devices.

## 1 Introduction

The multilayer ceramic capacitor (MLCC) [1, 2] is the “workhorse of the electronic components industry” [3] and a pervasive and critical element for electrical decoupling, filtering, and many other functions in advanced devices. A modern cell phone incorporates approximately 1000 MLCCs, and an electric vehicle incorporates about 10,000 MLCCs [3, 4]; about three trillion MLCCs were manufactured in 2018 [4]. The external form factors of MLCCs are extremely small, ranging from millimeters in scale to smaller than 500 μm × 250 μm [4, 5]. Internally, an MLCC consists of stacked layers of dielectric polycrystalline ceramic material, usually barium titanate (BaTiO_3_)—the subject of this work, interdigitated with layers of conducting metal electrodes (usually nickel) [1, 2]. The polycrystalline ceramic grain size is typically hundreds of nanometers [6], and the ceramic layers are typically tens of micrometers thick separated by micrometer-scale electrodes [6]. Up to 1000 layers may comprise a single MLCC [5], depending on capacitance requirements.

The manufacturing yield and operational reliability of an MLCC are predominantly determined by three phenomena effective at three different length scales: (1) At the millimeter scale of the MLCC, primarily affecting device yield and primarily mechanical in origin, MLCCs may be stressed and fracture through attachment to, or flexing of, the host printed circuit board [7]. (2) At the 10 μm scale of the dielectric layers, primarily affecting MLCC reliability in early use and primarily electrical in origin, the ceramic may break down at an electrical or thermal “hot spot” or defect, leading to an electrical short [8, 9]. (3) Finally, at the sub-micrometer scale of the grains, primarily affecting MLCC reliability in extended use and originating in mechanical and electrical coupling, the ceramic may lose its dielectric response, leading to a loss of component capacitance and thus device performance [1, 10]. Of these three phenomena, the first two are extrinsic to BaTiO_3_, and their prevalence can be reduced through improved manufacturing to reduce circuit board flexure and the number of breakdown-related defects. However, the third phenomenon is intrinsic to BaTiO_3_ and related dielectrics. Specifically, on cooling a polycrystalline dielectric during MLCC fabrication, the crystallographic structure of BaTiO_3_ leads to “locked-in” grain-scale residual stresses. These stresses influence subsequent microstructural development, including the initiation of microcracks, and, often more practically, impede the ability of the material to alter polarization in response to a changing electric field. Thus, improving the short-term yield and long-term electrical capacitance and reliability of MLCCs requires knowledge of the internal residual stress state and how that stress depends on underlying structure. Mapping of stress in BaTiO_3_ at the microscale is the subject of the current work.

In a recent series of studies [11–13], spatial variations of deformation and stress in single-crystal–based structures of BaTiO_3_ were mapped using electron backscatter diffraction (EBSD) techniques in a scanning electron microscope (SEM). At room temperature, BaTiO_3_ has a tetragonal unit cell [14, 15] with *a* and *c* axes. The unit-cell [001] *c* dimension is about 1% relatively longer than the [100] and [010] *a* dimensions, and the *c* axis is the direction of an electrical dipole moment. As a consequence, to minimize elastostatic and electrostatic energy [16], crystals of BaTiO_3_ usually divide into regions—known as domains—of differing unit cell orientation and polarization. The domain orientations are often related by symmetry, and particularly important domain boundaries are those at which the unit cells change orientation by nearly 90° and 180°. The early work of Bradt and Ansell [17–20] showed that electrical aging in BaTiO_3_ was due to the gradual formation of 90° domains as materials, particularly polycrystals, minimized internal stress in the tetragonal state. Later, the work of Arlt *et al*. [16, 21] showed that polycrystal grain-size effects in BaTiO_3_ were also due to 90° domain formation to minimize internal stress. Three-dimensional modeling of single crystals also showed the critical importance of electromechanical coupling in establishing domain structures [22, 23]. More recent works [10, 24, 25] have shown the direct effects of externally applied stress on both electrical aging and 90° domain formation and alignment, further supporting the view that device *electrical* behavior reflects the material *mechanical* stress state (and showing that aging is probably not a chemical effect, as suggested very early [26]). The mechanical variations noted in the studies above [11–13] arose in multidomain structures in which the *a* and *c* domain axes alternated in orientation perpendicular to the crystal sample surface, termed the *a* and *c* domains, respectively. Two structures were studied: The first structure consisted of large lamellar domains up to 10 μm wide extending through a millimeter-scale crystal [11, 13]. The second structure consisted of smaller domains about 1 μm wide in bundles terminating in a similarly sized crystal [12, 13]. As well as providing fundamental insights into the microstructure of BaTiO_3_, these studies demonstrated the power of EBSD to generate two-dimensional (2-D) maps of near-surface stress, strain, and rotation with sufficient resolution for application to MLCC structures, *i.e.*, about 10^−4^ resolution in strain and rotation with conjugate stress resolution of about 10 MPa, and lateral spatial resolution of about 50 nm. However, the studies were restricted to pseudo-periodic structures large relative to MLCC grain sizes.

Here, extending the previous studies, isolated domains, comparable in size to MLCC grains, were examined by EBSD in the SEM. This article begins with a brief overview of the materials and methods, noting the similarities to those used previously and two major differences: (1) The method was applied at a much smaller scale, and (2) individual isolated microdomains were examined. Strain results are then presented as previously (also in the Appendix), but the emphasis here is on stress, leading to presentation in principal coordinates and enabling comparison with the similarly measured stress field associated with an isolated micro-indentation [27, 28] and insight into possible fracture paths. A discussion follows the previous philosophy of inferring subsurface domain structure from the experimental constraint of surface-based EBSD rotation measurements. The importance of dislocations at domain terminations is emphasized.

To provide context, the work here forms part of an extensive set of techniques developed at the National Institute of Standards and Technology (NIST) to map stress: Neutron diffraction at centimeter length scales is used to map stress in large metal structures such as railway tracks [29]; X-ray diffraction at millimeter length scales is used to map stress in metal sheets for automobile applications [30] and at micrometer length scales to map stress in metal lines for microelectronic applications [31]; optical fluorescence techniques at sub-micrometer length scales are used to map stress distributions in polycrystalline ceramics to improve fracture resistance [32]; optical Raman spectroscopy techniques, also at sub-micrometer length scales, are used to map stress and strain distributions in single-crystal [27, 28] and polycrystalline silicon (Si) [33] for microelectromechanical systems (MEMS) applications; and electron diffraction (EBSD) techniques are used at the near-nanometer scale to map stress, stain, and rotation in Si [34] and SiGe alloys for microelectronic applications [35].

## 2 Materials and Methods

### 2.1 Materials

**Fig. 1:**
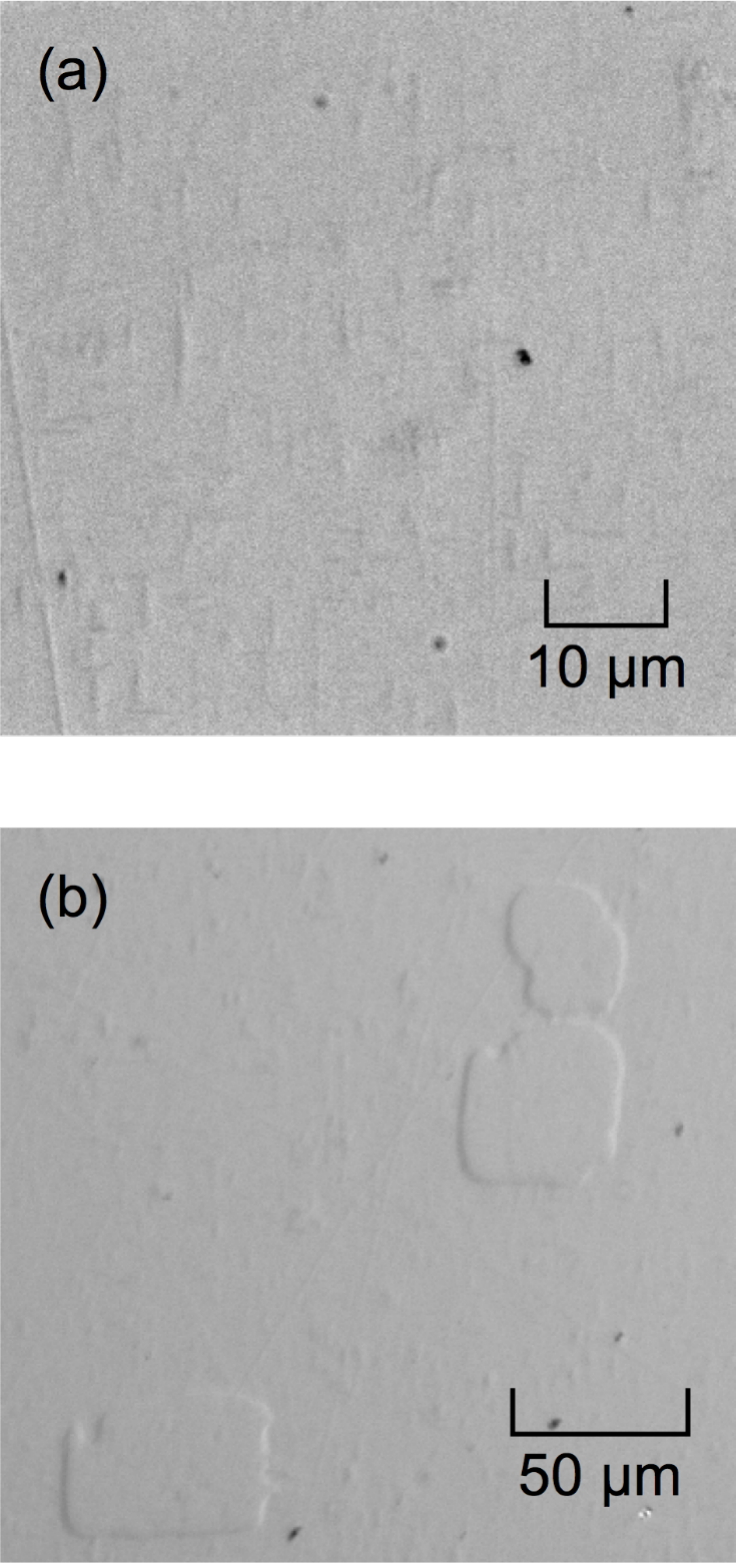
Optical micrographs of isolated domains visible on the surface of a BaTiO_3_ single crystal with majority domain polar *c* axis oriented perpendicular to and out of the image plane. Surface topography arises from differential etching of different domain orientations. (a) Isolated lenticular *a* domains with polar axis oriented parallel to the image plane and 90° domain boundaries with the majority domain. (b) Isolated blocky *c* domains with polar axis oriented into the image plane and 180° domain boundaries with the majority domain.

The sample for all experiments here was a BaTiO_3_ single crystal formed by the top seeded solution growth method (MSE Supplies, Tucson, AZ) and used in previous strain and rotation studies of bundled domains [12, 13].^1^1 Certain commercial equipment, instruments, and software are identified in this paper in order to specify the experimental procedure adequately. Such identification does not imply recommendation or endorsement by the National Institute of Standards and Technology, nor does it imply that the equipment or software identified is necessarily the best available for the purpose. The as-received sample was a plate, 5 mm × 5 mm × 1 mm, and nominally [100] × [010] × [001], electrically poled such that the majority of the sample was a single *c* domain, *i.e.*, a domain with *c* axis perpendicular to the large faces. A single large face of the sample as received was prepared by chemical-mechanical polishing. An additional polishing step using colloidal silica for 5 min was performed on this face. Optical micrographs of two regions of the polished surface are shown in Fig. 1. Figure 1(a) shows a set of features that appeared over the entire sample surface. These features were lenticular, ranging in length from (1 to 10) μm and width from (0.1 to 2) μm, and they exhibited a slightly raised topography with long dimensions parallel to the sample (and micrograph) edge. As will be shown below, these features are isolated 90° domains with the *c* axis nearly parallel to the surface and an *a* axis perpendicular to the surface and will be referred to as isolated *a* domains. Such surface features are not often reported, although isolated bulk *a*-*c* domain structures are common, and surface steps associated with bulk 90° domain boundaries (and their removal by stress) are apparently usual in crystal growth studies [36, 37]. Similar features have recently been observed on lead zirconate titanate [38]. Figure 1(b) shows a second set of features that appeared infrequently over the sample surface. These features were in the form of blocky, almost-square, mesas with somewhat irregular (10 to 50) μm edges parallel to the sample (and micrograph) edges, and they exhibited significantly raised topography relative to the surrounding matrix. The size, shape, and topography suggest these features are domains of reversed *c*-axis polarity, separated from the matrix by 180° domain boundaries. These types of domains have been reported often since the earliest BaTiO_3_ studies, especially the irregularly shaped boundaries [39–49], which are sometimes described as exhibiting a “watermark” geometry [42, 45–50]. These features will be referred to as isolated *c* domains. For both isolated *a* and *c* domains, the chemical-mechanical and colloidal silica polishing steps described above caused differential etching of the different domain polarization orientations on the surface, giving rise to the observed topography. The clearly raised nature in Fig. 1(b) suggests the features are negative polarization *c* domains in a positive *c* matrix [39, 40, 43, 47, 50–52]. The slightly raised topography of the isolated *a* domains in Fig. 1(a), less than that of the isolated *c* domains, is consistent with neutral *a*-domain etching intermediate to positive and negative *c*-domain etching [40].

**Fig. 2:**
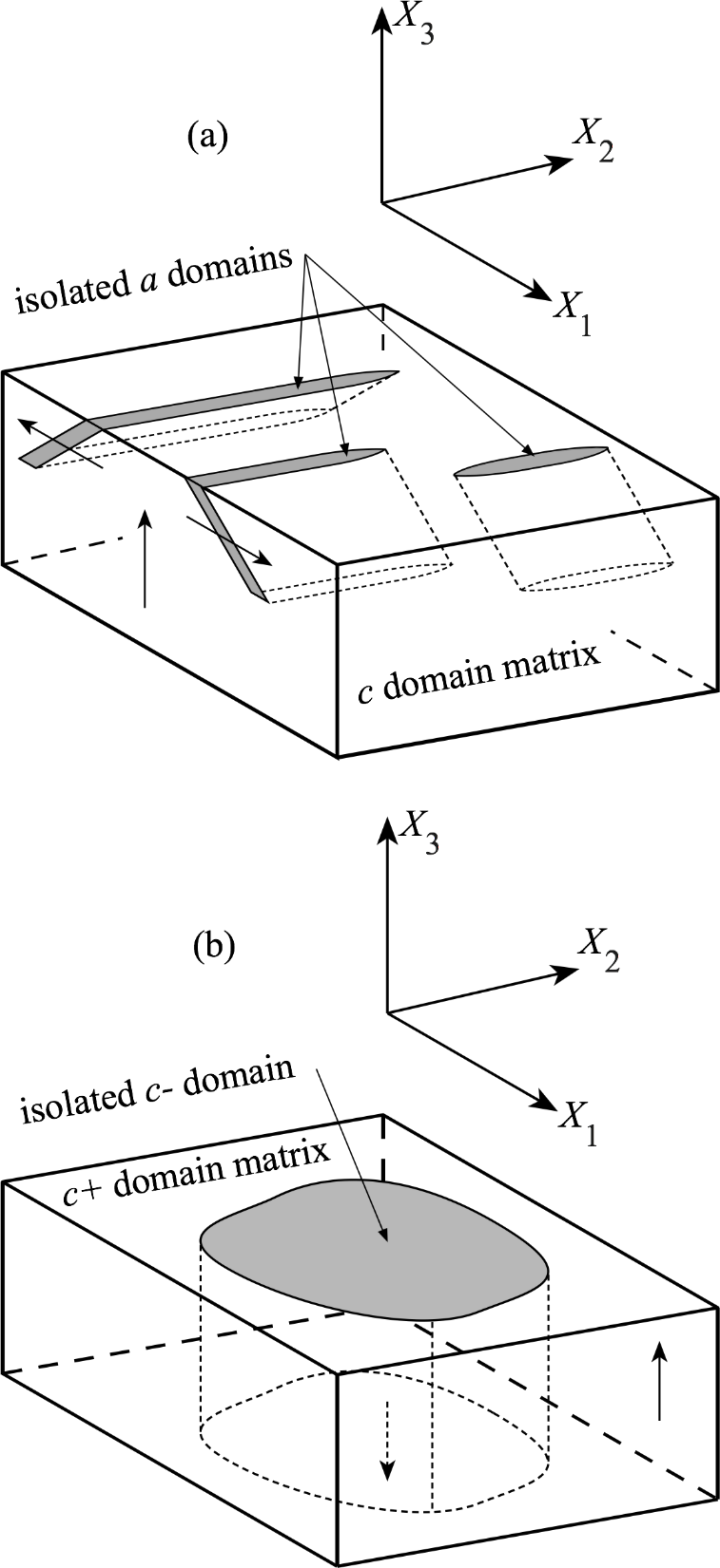
Schematic diagrams of isolated domains in a *c*+-domain BaTiO_3_ crystal. The surface planes are shown shaded, and the subsurface profiles are shown dashed. The dielectric polarization directions relative to the *X*_1_-*X*_2_-*X*_3_ sample axes are indicated by arrows. (a) Isolated *a* domains terminating subsurface. (b) Isolated *c*^−^ domain extending through the sample.

Schematic diagrams of the isolated domains and the *X*_1_-*X*_2_-*X*_3_ sample coordinate system are shown in Fig. 2, such that the *X*_1_-*X*_2_ planes are the image planes of Fig. 1. The arrows indicate the orientations of crystallographic [001] *c* axes. Figure 2(a) shows isolated *a* domains with extended surface dimensions aligned parallel to *X*_2_ in the *X*_1_-*X*_2_ plane. The perpendicular plane, *X*_1_-*X*_3_, has frequently been reported as the image plane in many optical and transmission electron microscope (TEM) studies of isolated *a* domains. The *X*_1_-*X*_3_ subsurface cross section of Fig. 2(a) shows the commonly observed crystallographic [101] and [$\overset{-}{1}01$] domain boundary orientations bisecting *X*_1_ and *X*_3_, with the domains terminating in the subsurface in wedge-shaped formations [17–19, 42, 45, 46, 48, 53, 54]. Figure 2(b) shows an isolated c^−^ domain in a c^+^ matrix with edges approximately aligned with the sample *X*_1_ and *X*_2_ edges. The subsurface extensions in the *X*_3_ direction of reversed *c* domains through the thickness of samples have been observed many times, including the earliest studies [31, 33, 46] and more recent digital reconstructions [55, 56]. A combination of EBSD orientation analysis and simulation has recently demonstrated images of reversed *c* domains in lithium niobate [57].

Many isolated domains were examined using the orientation and strain analyses described below, and representative results from a single isolated *a* domain and a single isolated *c* domain are presented herein.

### 2.2 Orientation and Deformation Analysis

The orientation and deformation of the sample matrix and domains were determined from electron backscatter patterns (EBSPs) obtained from the polished sample surface. The surface was not coated prior to loading into a field emission SEM (Hitachi S4700 FESEM, Hitachi High Tech, Tokyo, Japan) for analysis. High-resolution EBSPs were recorded using an accelerating voltage of 20 kV and a beam current of ≈ 2 nA with the sample normal tilted 70° about *X*_1_ relative to the SEM electron beam. Asymmetric 2-D grids of EBSPs were collected from the *X*_1_-*X*_2_ plane. For the *a* domain, the grid was 175 × 20 steps in the *X*_1_ × *X*_2_ directions, respectively, generating 3500 EBSPs from points separated by 30 nm in the *X*_1_ direction and 250 nm in the *X*_2_ direction, producing high-resolution maps approximately 5.25 μm × 4.75 μm. For the *c* domains, the grid was 150 × 17, generating 2550 EBSPs separated in the *X*_1_ direction by 400 nm and in the *X*_2_ direction by 2.8 μm, and creating 60 μm × 48 μm maps. Each EBSP consisted of an image of 1344 × 1024 pixels; no binning was applied to the EBSPs, which were recorded at high gain with automatic and static background correction. Each EBSP was collected in approximately 1 s and was of sufficiently high quality such that each EBSP could be indexed to obtain crystal orientation at a scan point using Oxford HKL Flamenco software (version 5.0.9.1, Oxford Instruments, Abingdon, U.K.). Detection of the edges of Kikuchi bands in a circular region (radius = 511 pixels) centered on the middle of an EBSP was performed using a Hough transform method operating at maximum achievable resolution. Indexing was determined from the automatic detection of five to six bands and provided the local orientation of the tetragonal (*a*, *a*, *c*) crystal axes relative to the *X*_1_-*X*_2_-*X*_3_ sample axes in terms of Euler angles. Two domain types were observed. A local crystal orientation in the sample was regarded as part of a *c* domain if the (*c*, *X*_3_) angle was close to 0, and as part of an *a* domain if an (*a*, *X*_3_) angle was close to 0. In practice, (*a*, *X*_2_) angles were always close to 0, such that the transformation from *c* domain to *a* domain was accomplished by ± 90° rotation about *X*_2_.

The grid of EBSPs was analyzed to generate stress, strain, and rotation maps using the method of cross-correlation (CrossCourt 3.0, BLG Productions, Bristol, U.K.) [58–60]. Reference patterns from points in each data set were assigned (by definition) zero strain and rotation, and all deformation is thus relative to these points. Two reference patterns were used, one in the center of a *c* domain and one in the center of an *a* domain (domains were determined from the orientation analysis); all *c* domains were analyzed with the *c*-domain reference, and all *a* domains were analyzed with the *a*-domain reference. The details of deformation analyses by cross-correlation of EBSPs and extensive application to BaTiO_3_ are considered elsewhere [11–13, 58–60]. Briefly, the eight independent components of the traceless distortion tensor in crystal coordinates were determined at each grid location from analysis of 20 regions of interest (256 pixels × 256 pixels) from each EBSP. To obtain the full nine-component distortion tensor, *A_ij_*, the *X*_1_-*X*_2_ surface was considered to be in mechanical equilibrium and normal traction-free, such that the closure condition $\sigma_{33}=0$ was imposed at all locations. No additional flattening or background subtraction of *A_ij_* was required or imposed [27, 28]. The local infinitesimal strain tensor, ε*_ij_*, was obtained as the symmetric component of the full distortion tensor, $\varepsilon_{ij}=(A_{ij}+A_{ji})/2$. The local infinitesimal rotation tensor, ω*_ij_*, was obtained as the antisymmetric component of the full distortion tensor, $\omega_{ij}=(A_{ij}-A_{ji})/2$, noting that the closure relation is not required in this case. The polar rotation tensor, $\theta_{ij}$, using a single *c*-domain reference [13] was not used ($\theta_{ij}=\omega_{ij}$, except for *a* domains $\theta_{13}={-\theta }_{31}=\omega_{13}-\pi /2$). The stress, σ*_ij_*, was obtained from strain using σ*_ij_* = *c_ijkl_*ε*_kl_* and the appropriately rotated domain-dependent elastic constants *c_ijkl_* [11, 12]. Global mechanical equilibrium was imposed by adjusting individual stress components such that the resultants had mean values of zero, noting that the condition $\sigma_{33}$ = 0 was automatically fulfilled [12]. The values and orientations of the principal stresses were determined using conventional methods [61].

## 3 Results

Optical microscopy, SEM, and EBSD revealed the domain structure, surface strains, and surface stresses. The domains observed here differed from earlier work in that they were relatively isolated, although they exhibited similar patterns of crystal rotation. Stress and strain maps exhibited large spatial variations and correlated with the measured domain structure. Each of these aspects is examined in turn.

### 3.1 Structure

**Fig. 3:**
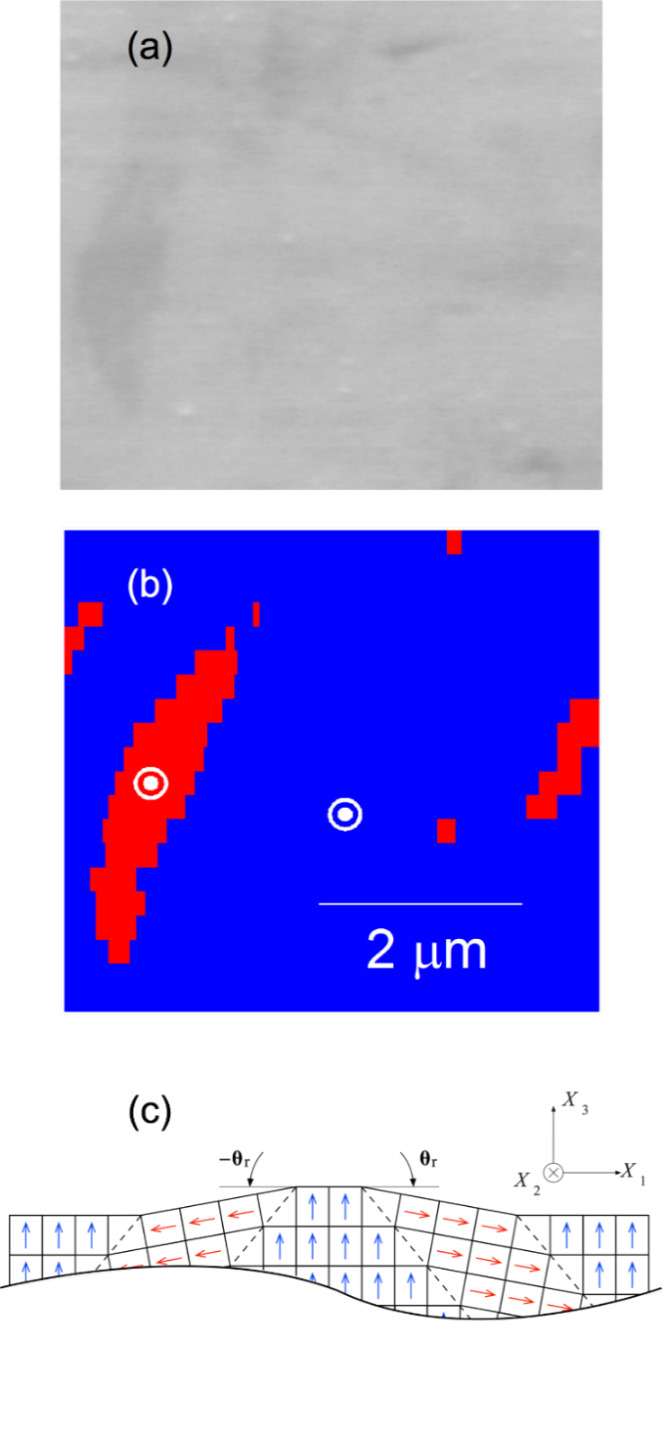
Detailed views of isolated *a* domains in a *c*-domain BaTiO_3_ matrix. (a) SEM image of the *X*_1_-*X*_2_ surface in Fig. 2 showing weakly contrasting domain on the left (secondary electrons). (b) Domain orientation index map of same area as (a) showing clear *a* domain on left, extending primarily parallel to the *X*_2_ direction. The locations of the (assigned strain-free) reference points are indicated by the bull’s-eye symbols (backscattered electrons). (c) Schematic cross section of the *X*_1_-*X*_3_ surface (not to same scale as [b]).

Figure 3(a) is a SEM image of a 5.25 μm × 4.75 μm isolated domain region on the sample. The contrast of the domains is weak relative to the optical image of Fig. 1(a), and the topography is not very evident. Nevertheless, a dark elongated feature is visible on the left of the image, and a less distinct feature is visible on the right. Much greater contrast is visible in the EBSD index map of the same region in Fig. 3(b), which shows the pixels indexed as “*a*” in red on a blue “*c*” field. There is indeed a large elongated *a* domain in the center left and smaller *a* domains to the center right and perhaps upper left. Figure 3(c) is a cross-sectional schematic diagram of the *c*-domain field with two isolated *a* domains, complementing the plan-view observations of Fig. 3, parts (a) and (b). The schematic cross section is consistent with the orientation variation measurement of Fig. 3(b) and a rigid rotation description of domain boundary structure [13]. In this description, domain boundary unit cells are deformed and exhibit diagonal mirror symmetry, and cells in domains on either side of a boundary are undeformed and related by rotation. The domain boundaries are approximately parallel to *c*{101} planes and are thus here inclined to both *X*_3_ and *X*_1_; see Fig. 2(a). The unit cell *c*/*a* ratio of tetragonal BaTiO_3_ requires the scalar rigid rotation angle, *θ*_r_, to be 0.011 rad (or about 0.63°); see Fig. 3(c) [14, 15]. In addition, charge neutrality requires the unit-cell ferroelectric dipoles to maintain relative “head-to-tail” orientations across domain boundaries. Arrows indicate dipole orientations, and dashed lines indicate domain boundary cells with indefinite dipole character. For visualization purposes, the BaTiO_3_ tetragonal distortion and domain rotation angle are exaggerated by a factor of 20.

The domain and domain boundary sequence from left to right in Fig. 3(c), using previous notation [13], is *c*^+^//*a*^−^//*c*^+^//*a*^+^//*c*^+^, in which a letter identifies the domain, a superscript represents the polarization relative to *X*_3_ or *X*_1_ as appropriate, and // represents a domain boundary. Boundary orientation is constrained by the specified surface orientation and polarization. The right-hand sequence in Fig. 3(c), *c*^+^//*a*^+^//*c*^+^, positive *θ*_r_, is often depicted (see [13] for a list), and the domain boundaries in this case proceed downward to the right. The left-hand sequence, *c*^+^//*a*^−^//*c*^+^, negative *θ*_r_, is less often depicted (*e.g*., [15]), and, in this case, the domain boundaries proceed downward to the left. In both cases, a key assumption is that the *c* domains are not connected beneath the surface and are completely separated by an intervening *a* domain such that the structures of Fig. 3(c) may be regarded as semi-infinite in *X*_3_. This assumption is reasonable in the case of large lamellar *a* domains that extend though a sample [11], and Fig. 3(c) is thus, in this case, an accurate representation of the entire domain structure. In the case of small *a* domains, either bundled [12, 13] or isolated as here (Fig. 3[b]), it is probable that the *a* domains are finite and terminate in the subsurface (Fig. 2[a]), such that Fig. 3(c) represents only the surface domain structure. Isolated domain termination structure is considered further in the Discussion.

**Fig. 4:**
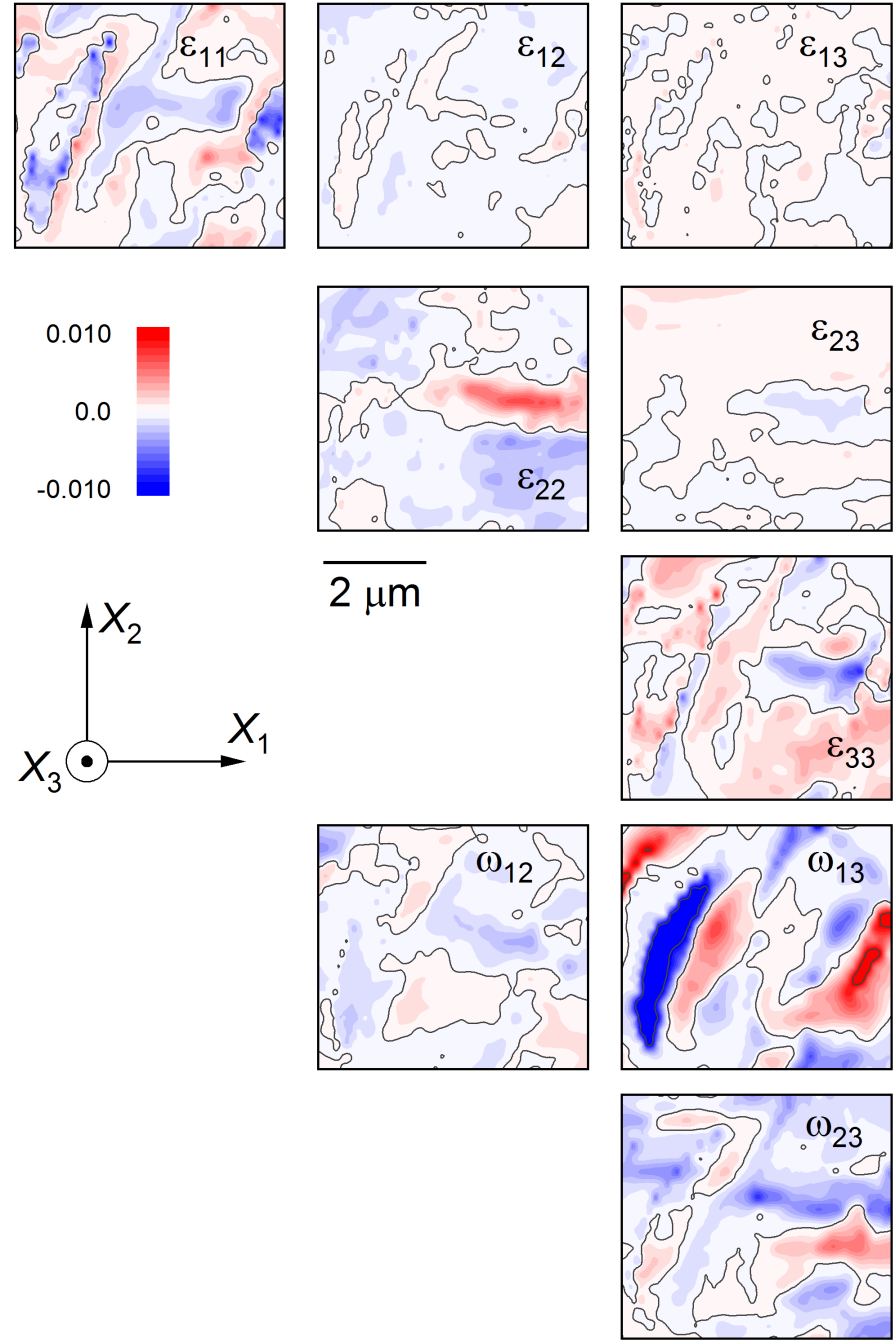
Deformation of isolated domain region shown in Fig. 3 represented as color-filled contour maps of the strain and rotation tensor components in indicated sample coordinates: Strain, ε*_ij_*, symmetric; and rotation, ω*_ij_*, antisymmetric.

### 3.2 Strain

Figure 4 shows the region of Fig. 3(b) as a “visual tensor” representation of deformation in the form of color-filled contour maps of the six independent components of *ε_ij_* (symmetric) and three independent components of *ω_ij_* (antisymmetric). Such representation was used previously for indentations and MEMS structures in silicon [28, 34, 59, 60]. The contour range for all maps is ± [(*c*/a) −1], ≈ ± 0.01, which is the relative tetragonal distortion of BaTiO_3_ and the rigid-domain rotation angle (in radians, see above); the scale and axis orientation are indicated. Perhaps the most striking feature of Fig. 4 is that the deformation is dominated by the *ω*_13_ rotation component. There is a distinct negative rotation region localized *on* the large *a* domain on the left (blue). In addition, there are large positive rotations localized on the smaller *a* domains to the right and extreme left, and a weak positive rotation adjacent to the large domain (all red). The *ω*_12_ and *ω*_23_ rotation components exhibit few distinct features. Conversely, the major feature in the strain field is the distinct positive *ε*_22_ region in the *c* domain *between* the *a* domains in the center of the image (red). In addition, there is a negative *ε*_11_ region localized on the *a* domain to the left and a negative *ε*_33_ region between the *a* domains (both blue). In these laboratory Cartesian coordinates, the *ε*_12_, *ε*_23_, and *ε*_13_ shear strain components exhibit few distinct features, and the strain field is dominated by the normal strains. More detail and consideration of the strain and associated rotation variation about the isolated *a* domain are given in the Appendix, noting the similarities with the lamellar domains observed previously [11–13].

### 3.3 Stress

**Fig. 5:**
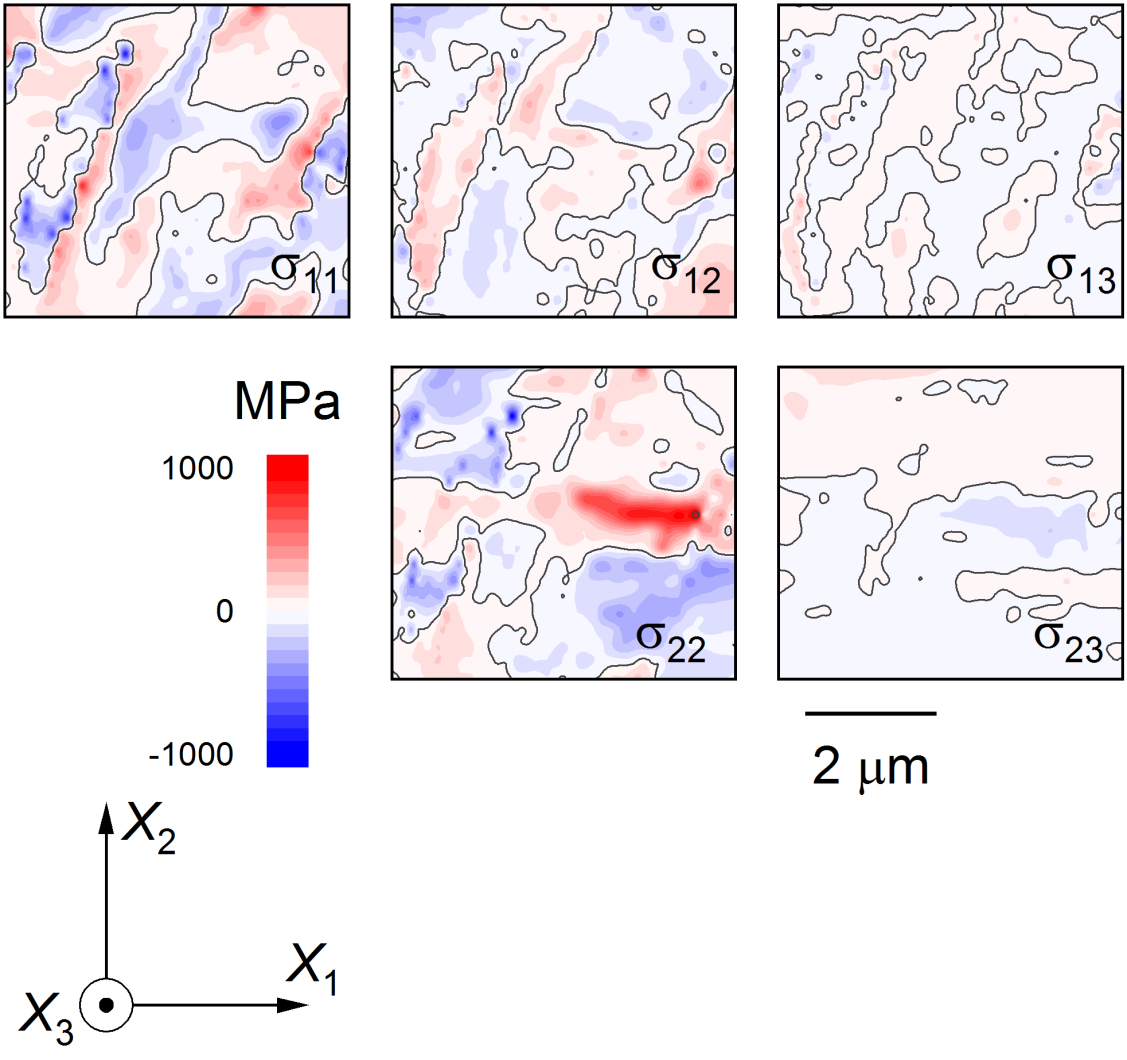
Stress tensor components of isolated *a*-domain region from Figs. 3 and 4 represented as color-filled contour maps in indicated sample coordinates.

**Fig. 6:**
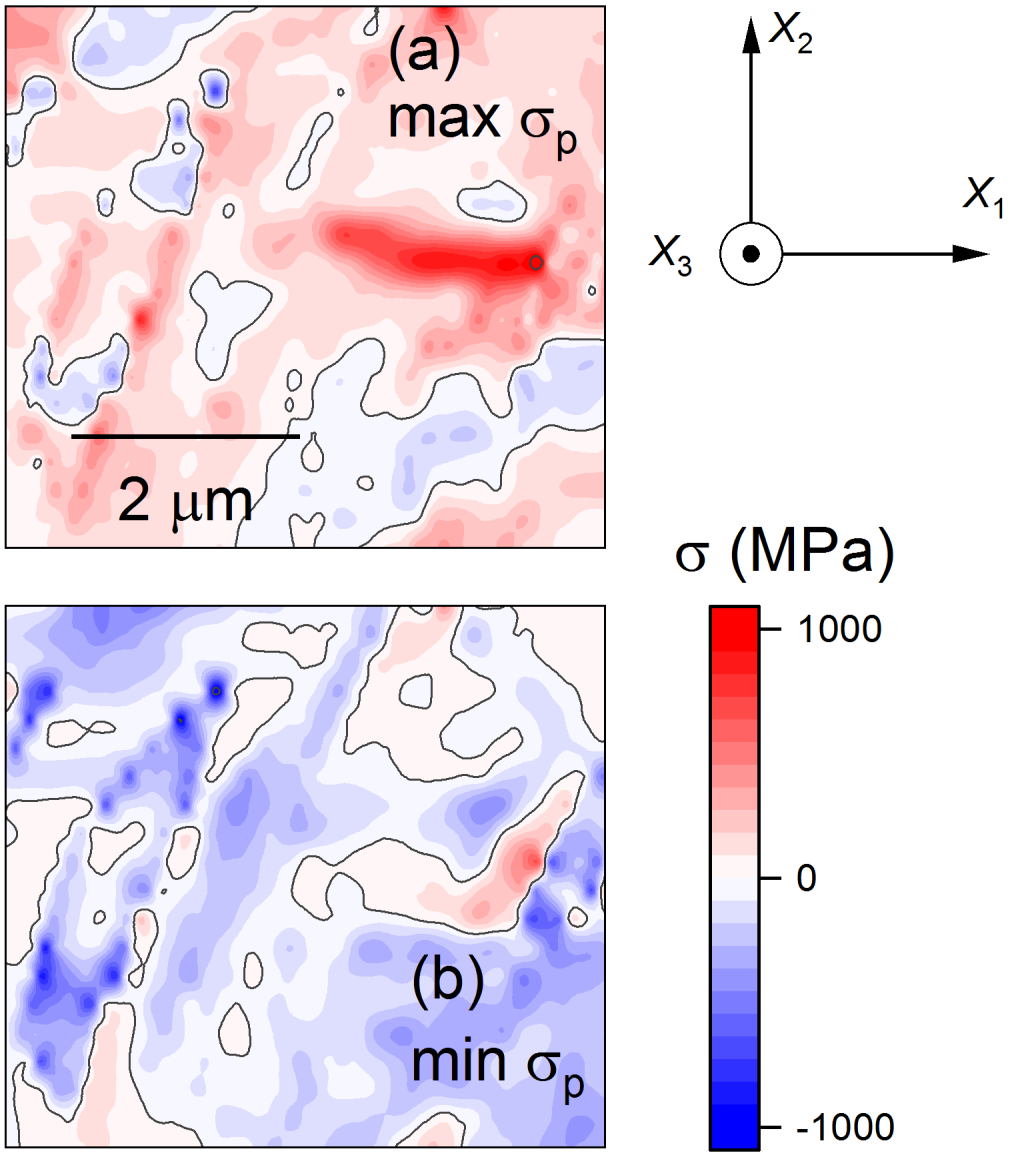
Stress of isolated domain region shown in Figs. 3 and 4 represented as color-filled contour maps of the principal tensor components. Sample coordinates are indicated. (a) Maximum principal stress, max σ_p_. (b) Minimum principal stress, min σ_p_.

As demonstrated previously for lamellar and bundled domains [11, 12], stress component maps using the laboratory Cartesian axes were generated from strain data using the elastic constants of BaTiO_3_. For the isolated domains here, a visual representation of the stress tensor in Cartesian axes was generated, shown in Fig. 5, noting that σ_33_ = 0 is a boundary condition and is absent. The Cartesian stress components of Fig. 5 strongly resemble the conjugate Cartesian strain components of Fig. 4. Stress information that is unbiassed by the axes selected and that is more directly useful for interpreting potential fracture behavior can be obtained using principal coordinates [28]. Based on the Cartesian stress tensor data, the dominant in-plane principal stresses were determined, and the values are shown as color-filled contour maps in Fig. 6. Two features are quite apparent in these maps: The maximum principal stress, max σ_p_, shown in Fig. 6(a), consists of mostly positive values and is dominated by a lobe of tension in the *c* domain between the isolated *a* domains, in the center-right of the map. The minimum principal stress, min σ_p_, shown in Fig. 6(b), consists of mostly negative values, and the dominant feature here is the compression, localized within the large *a* domain on the left of the map. This compression within the domain is also visible in the max σ_p_ map along with tension on the domain boundary. The maps in principal coordinates of Fig. 6 make microstructural effects clear with no bias regarding feature or axis orientation.

**Fig. 7:**
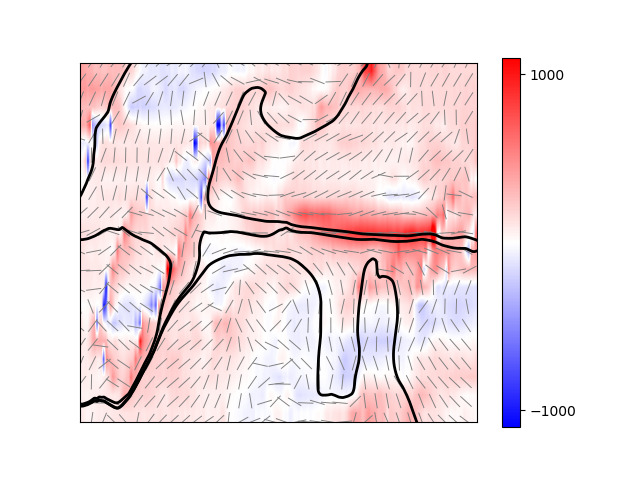
Principal stress map for isolated domain regions shown in Figs. 3, 4, and 7. Color-filled contours indicate maximum principal stress values in units of megapascals (MPa). Short fine lines indicate minimum principal stress directions (every fifth point shown). Elongated lines are minimum principal stress trajectories.

Information regarding the orientation of the principal stresses is shown on Fig. 7, which consists of several layers. First, the local values of max σ_p_ are shown as a color-filled contour map, as in Fig. 6(a). Second, the local orientations of min σ_p_ are shown as the short fine lines (for clarity, every fifth orientation is shown). Third, the minimum principal stress trajectories are shown as the continuous bold lines. These trajectories were obtained by (numerically) integrating the differential equation implicit in the determination of the principal stresses. The minimum principal stress trajectories in Fig. 7 are perpendicular to the local maximum principal stresses and as such provide estimates of potential fracture paths [62]. A feature apparent in Fig. 7 is that the trajectories tend to converge in two areas: in the region between the domains and along the edge of the large domain. The implication, given that these are also regions of tensile stress, is that these are areas of greater fracture probability and that cracks would preferentially propagate along these trajectories, somewhat independent of initiation location, leading to cracking in the *c* domain and delamination of the *a* domain from the *c* domain.

In more detail, fracture of single-crystal BaTiO_3_ is characterized by a toughness of 0.7 MPa m^1/2^ [12], representing the equilibrium resistance to crack propagation. The relevant driving force for fracture is the stress-intensity factor, for which the value and variation can be obtained explicitly by integrating a known stress field over an existing or candidate crack path, as demonstrated previously for a BaTiO_3_ microstructure [12]. Such an integration was not performed here, but estimates from Fig. 7 suggest a tensile stress zone of about 1 GPa characteristic magnitude and about 1 μm extent, providing a characteristic crack driving force expressed of about 1 MPa m^1/2^. When compared with the toughness of BaTiO_3_, this value suggests that cracks 1 μm in length would be in equilibrium in the BaTiO_3_ microstructure of Fig. 1, in the *c* domain between the *a* domains. The localized nature of the tensile stress fields along the trajectories in Fig. 7 suggests that such cracks would be in stable equilibrium as “microcracks” and, although perhaps degrading the electrical and stiffness properties of an MLCC, would not lead to catastrophic device failure. The subsequent superposition of a uniform stress by circuit board flexure after MLCC fabrication could propagate such cracks and lead to device fracture and failure [7]. These considerations depend on the microstructural stress state remaining relatively unaltered by the initiation of cracks or delamination. If such effects do alter the stress field, domain wall motion or other relaxation effects are then also possible. The large stresses observed here suggest that such sequential electromechanical effects could be important, and that other accommodation mechanisms, *e.g.*, formation of point defects or finite-width charged domain boundaries, which do not greatly alter the electrical response or mechanical compliance, may also operate.

An important final result is the strain field observed surrounding the very common *c* domains shown in Figs. 1(b) and 2(b). Consistent with the many previous observations [18, 31, 32, 37, 38, 42, 47, 49], no strain of any significance with any distinguishing features or correlation with optical or SEM images was observed at any *c* domain or, by implication, associated with any 180° domain boundary. (Strictly, this was observed for 180° boundaries parallel to the *c* polarizations in the separated domains. Strain has been observed at 180° boundaries tilted relative to *c* [63].) This important null result places the above *a*-domain observations in context and is supported by the strain tensor with domain overlay shown in the Appendix.

## 4 Discussion

The observations here have extended the application of EBSD to small-scale deformation measurements in BaTiO_3_. In a materials engineering sense, the demonstration of strain and rotation resolutions of approximately 10^−4^ with image pixel spacing of approximately 30 nm over maps about 5 μm square (Fig. 4) brings EBSD into the realm of commercial MLCC device dimensions. The use of such information to generate similarly sized maps of principal stresses and principal stress trajectories with resolutions of about 10 MPa (Figs. 5, 6, and 77) is of great practical importance for optimizing MLCC yield and reliability, which are currently limited by domain locking and cracking and fracture. In a materials science sense, study of isolated domains expands the number of different features examined in BaTiO_3_, enabling similarities and differences in underlying domain microstructure to be identified. Here, the similarities were noted in the strain variations within a single isolated domain that were comparable to those observed in lamellar [11] and bundled [12] domains. In particular, the strains suggested that unit cells within domains were more “cubic” at domain edges; *e.g*., *a*-domain tetragonal cells were compressed in plane and expanded out of plane to accommodate inclusion in a surrounding *c*-domain matrix (Fig. 4). Differences were noted in the rotation variation across multiple domains. In the lamellar and bundled structures, the rotation variations were all of one sign, suggesting that multiple lamellae and bundles generated macroscopic curvature of the single-crystal surface, an idea suggested in the earliest work [53]. Here, the rotation variations were of opposite signs, *e.g*., Fig. 4, suggesting that the isolated domains did not lead to surface curvature. The fundamental structure of BaTiO_3_, constrained by the isolated nature of the domains (Fig. 3), the localized rotation (Fig. 4), and the previous observations of subsurface termination [17–19, 42, 45, 46, 48, 53], are now considered.

The individual rotation and domain sequences in Fig. 3(c), 0//−*θ*_r_//0 and *c*^+^//*a*^−^//*c*^+^ (left) and 0//*θ*_r_//0 and *c*^+^//*a*^+^//*c*^+^ (right), are similar to those observed in previous investigations of large (> 10 µm) lamellar domains [11, 13]. Such lamellae were observed to extend completely through a millimeter-scale sample, and thus the sequences of Fig. 3(c) were considered to be adequate descriptions of the complete domain structure. The rotation and domain sequences of Fig. 3(c) are also similar to those that were most frequently observed in investigations of small (1 µm) domains in bundles [12, 13]. The bundles were not observed to extend through the sample, and hence it was inferred that the domains terminated in the subsurface. The inference was strengthened by the occasional observation of simultaneous 0//*θ*_r_//2*θ*_r_ and *c*^+^//*a*^+^//*c*^+^ sequences. These sequences provided a clear indication of subsurface intersecting domain boundaries and *a*-domain termination. The consequent implication was that the surrounding *c*-domain material was connected beneath the surface by a dislocation-mediated low-angle grain boundary [13]. In the case of the bundles, Fig. 3(c) was thus considered to be only a description of the domain surface structure. In the case here of ultrasmall (0.1 µm) isolated *a* domains remnant from the *c*-domain poling process, the rotation and domain sequences of Fig. 3(c) are also observed, similar to the lamellae and the bundles. However, as for the bundles but different from the lamellae, the isolated domains were not observed to extend through the sample. This observation and the small size again suggest subsurface termination, although with a different surface constraint and thus different termination structure from that considered earlier [13]. The probable structure is considered below and has implications not just for isolated domains but for the majority of bundled domains.

**Fig. 8:**
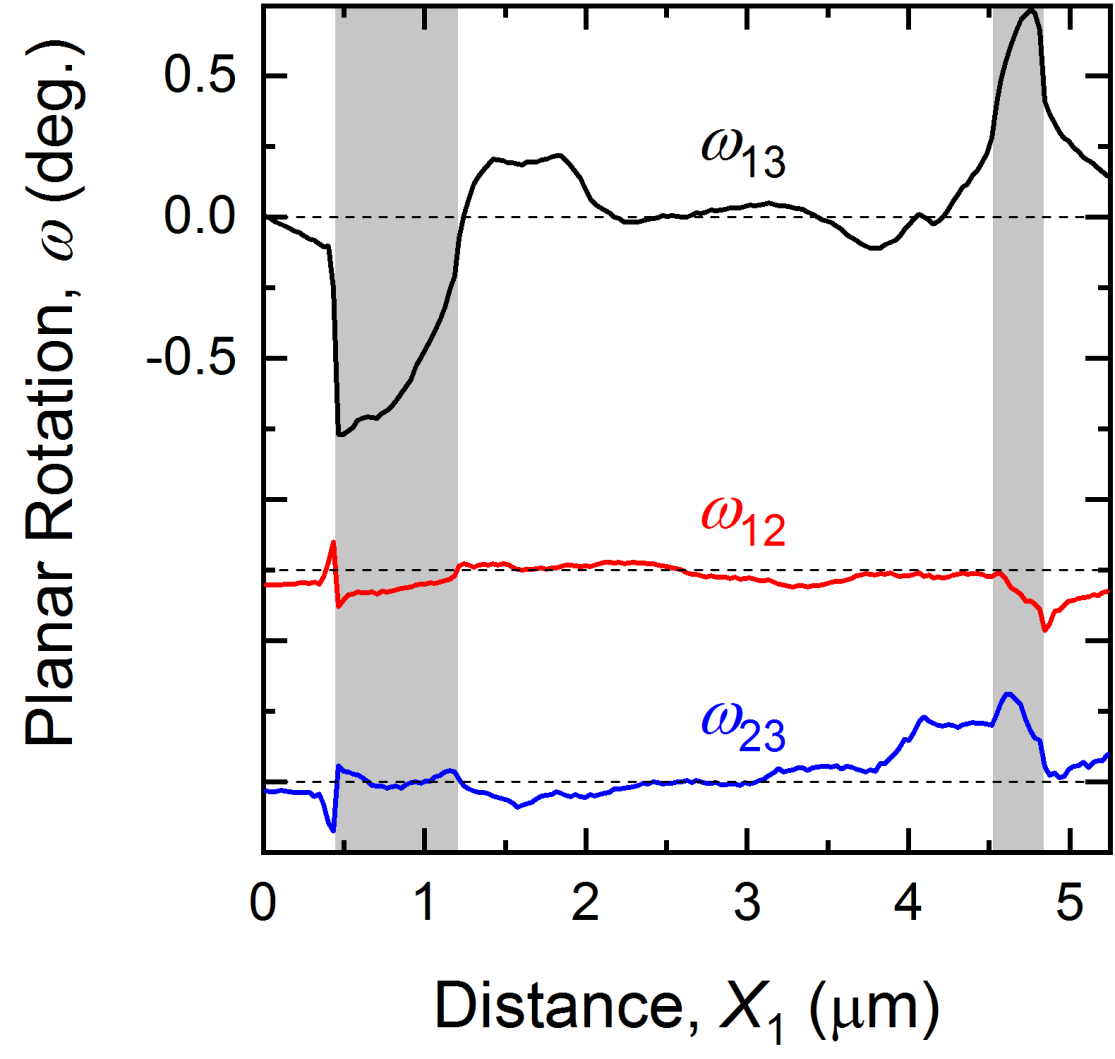
Line profiles of the rotations in the *X*_1_ direction across the center of the maps of Fig. 4. For clarity, the ω_12_, and ω_23_ data are offset from zero; the locations of zero rotation are indicated by dashed lines, and *a* domains are indicated by shading.

**Fig. 9:**
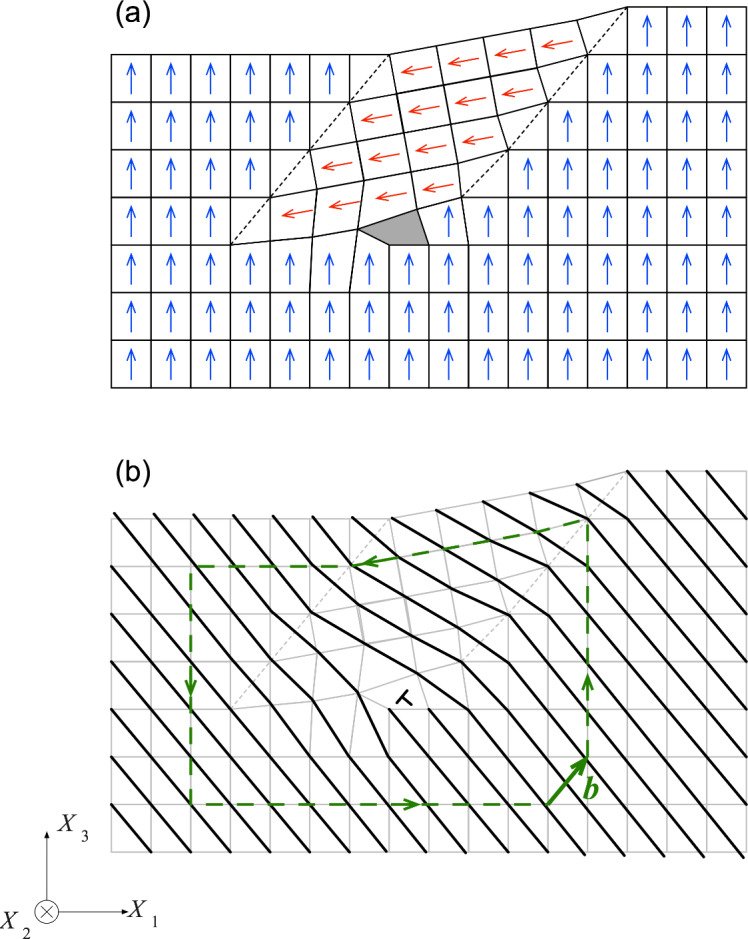
Schematic cross sections of an isolated *a* domain in a continuous *c*-domain matrix consistent with orientation indexing, surface rotation measurements, charge balance, and subsurface termination observations. (a) Domain and dipole diagram indicating 90° domain boundaries as dashed lines left and right and a dislocation core in the subsurface. (b) Dual lattice diagram indicating the dislocation Burgers vector inclined to both domains and the surface.

Perhaps the greatest constraint on inferred subsurface structure is surface rotation. Figure 8 shows line scans of rotations taken from the center of the maps in the *X*_1_ (horizontal) direction in Fig. 4. In contrast to Fig. 4, the rotations are expressed in degrees, noting that the rigid rotation angle as shown in Fig. 3(c) is about 0.63°. Figure 8 makes clear the greater magnitude of the *ω*_13_ rotation relative to the *ω*_12_ and *ω*_23_ rotations, the spatial localization of this rotation to the *a* domains, the reversal of sign from one *a* domain to the next, and the approximate agreement between the magnitudes of the peak values within the *a* domains with the rigid rotation value. The negative-zero-positive rotation sequence of *ω*_13_ in Fig. 8 also agrees very well with that shown in the schematic *a*^−^//*c*^+^//*a*^+^ central domain sequence in Fig. 3(c). Building on these agreements, and consistent with charge neutrality and domain indexing, Fig. 9(a) is a schematic cross-sectional diagram of an isolated *a* domain terminated in a *c*-domain field. The subsurface structure of the *a* domain in Fig. 9(a) was constructed as follows, as the simplest of many alternatives: A single undeformed *c* domain with a surface step of height one unit cell was considered as an initial configuration. An area of material on the surface of the domain at the step was removed as a discrete number of cells. The initial *c*-domain cells are indicated by blue arrows parallel to *X*_3_. A single *a* domain of 4 × 4 cells was rotated by −*θ*_r_ and inserted into the excised *c* domain. The initial *a*-domain cells are indicated by red arrows slightly inclined to *X*_1_. An ideal rigid rotation domain boundary was formed along the left edge of the *a* domain. A near-ideal boundary was formed along the right edge of the *a* domain by weakly deforming *a*-domain and boundary cells. Domain boundary walls are indicated by dashed lines.

The finite domain structure of Fig. 9(a) is perturbed from the semi-infinite structure of Fig. 3(c) due to the connected nature of the single *c*-domain matrix. The initial excised *c* domain included a step height that was filled by the inserted *a* domain: In the semi-infinite case, in which the *c* domains are unconnected, step height is determined solely by the assumed width of the intervening *a* domain. In the finite case here, step height was determined by a discrete number of *c*-domain cells, and weak deformation at the right boundary was required to accommodate the imposed *c*-domain geometry. A surface step is required by the *a*-domain rotation, and, as a consequence, deformation is required to maintain domain compatibility at the top surface. In contrast to the weak subsurface perturbation in Fig. 3(c), the subsurface structure of Fig. 9(a) is strongly perturbed by the imposition of compatibility at the *a*-domain termination. Cells in both the *a* domain and *c* domain were strongly deformed in Fig. 9(a) so as to form a continuous lattice at the subsurface domain boundary. In one area, lattice continuity could not be maintained, and this area is shown shaded in Fig. 9(a).

Inspection of Fig. 9(a) shows that the shaded area is the core of a dislocation with Burgers vector **b** = *a*[100] + *c*[001] and line direction perpendicular to the page (*X*_2_ direction). Figure 9(b) shows the same unit cells as in Fig. 9(a) but as light-gray lines. Diagonal dark lines indicate the traces of (101) planes and half-planes of unit cells. The dislocation consists of two terminating half-planes of unit cells (as opposed to half-planes of atoms as in metals: a consequence of charge neutrality). The Burgers vector is also demonstrated by considering the Burgers circuit, shown using the finish-start convention (dashed-line with arrows). The shown dislocation can also be decomposed into two orthogonal dislocations, **b** = **b**_1_ + **b**_2_, where **b**_1_ = *a*[100], and **b**_2_ = *c*[001], with one set of extra half-planes of unit cells below the dislocation and normal to the *X*_1_ direction, and a second set to the right of the dislocation and normal to the *X*_3_ direction.

Figure 9 is easily extended to isolated domains much larger in terms of the number of cells involved and in which the surface steps are larger, perhaps visible as in crystal growth studies [36, 37]. In those cases, although the left and right domain boundaries remain unaltered, the subsurface termination takes on a serrated form consisting of repeated instances of Fig. 9 and a periodic array of dislocations. The dislocation array spacing is entirely set by the angular deviation of the *a* domain from the *c*-domain matrix. The number of dislocations, *N*, is set by the size of the isolated domain and can be approximated by *N* ≈ *θ*_r_*l*/*c*, where *l* is the domain width. For the left domain in Fig. 3(b), *l* ≈ 0.7 μm, and for BaTiO_3_ [14], *c* ≈ 0.4 nm, and *θ*_r_ ≈ 10 mrad, such that *N* ≈ 20, and the total Burgers vector for the isolated domain is *N***b** ≈ *c*[20 0 20]. This surface inclusion domain structure, delimited by parallel rotation domain boundaries, should be compared with a similar structure delimited by intersecting domain boundaries studied earlier [13]. In the earlier case, the terminated *a* domain was accommodated by tilt in the *c*-domain matrix, modelled as a low-angle grain boundary and periodic array of edge dislocations. Once again, dislocation spacing was set by angular deviation of the *a* domain from the *c*-domain matrix, but the Burgers vector of these dislocations was approximately **b** = *a*[100], and the number of dislocations was set by the thickness of the sample.

It is clear that subsurface termination of domains within differently oriented matrices requires accommodating dislocations to maintain structural compatibility. Recent observations [64] using dark-field X-ray microscopy of a lamellar domain array in a *c*-domain crystal indicated $\langle 110\rangle$ trace orientations, subsurface terminations, and curved domain boundaries, probably accommodated by dislocations, with extended strain fields, all as inferred here from EBSD. For bundled domains, many dislocations would be required to accommodate the multiple changes in orientation, and hence a prediction from the above considerations is that the surfaces of bundles should be rumpled or rough, and this is borne out by experimental observation [12, 64]. In addition, for both bundled and isolated domains, the stress and strain fields associated with dislocations should exist within domains and extend well beyond domain boundaries, and this is also borne out by observation. An interesting engineering question is whether, during an electrical poling operation, isolated and bundled domains form as a consequence of dislocation-based domain “pinning” or dislocations form as a consequence of incomplete poling, leaving isolated or bundled domains. Termination of domain boundaries may also be accommodated by charged domain boundaries as reviewed extensively elsewhere [65, 66]. Such boundaries remove the strict “head-to-tail” domain boundary polarization requirement and allow the formation of local non-dislocation structures to accommodate domain terminations and rotations, but they do not remove all surface “rumpling” [66]. Electrical boundary conditions are likely to be critical to the formation of such boundaries, and thus the removal of dislocations, as free-charge carriers are required for screening charged features, providing even greater electromechanical coupling.

Longer-range behavior is highlighted in Fig. 8, which shows peaked variations in *ω*_13_ extending over approximately 1 μm on the left and 0.5 μm on the right, somewhat inconsistent with the abrupt, stepped variation implied by Fig. 3(c). Two possible explanations for this difference are that (1) the enclosed *a* domain is not formed by rigid rotation but by gradual rotation, and Fig. 3(c) is only approximately correct, or (2) the information volume of the EBSD probe convolutes a range of rotations, both laterally and in depth, and Fig. 8 only approximates surface rotations. These are experimental questions to be addressed by further stress mapping observations or possibly modeling or simulations of the long-range elastic distortions and stresses. The idealized domain walls depicted in Fig. 3 and in many prior works give rise to charge-neutral domain walls and stress-free rigid rotations of *a* domains with respect to *c* domains, contrary to observation. Similarly, the Ginzburg-Landau-Devonshire (GLD) model, which even takes into account finite domain wall thickness, predicts that stresses and strains are localized to the nanoscale region around the domain wall [67]. However, the boundary conditions in the present work are different. Most notably, the GLD-simulated domain wall was not oblique to a free surface, differing from the present case. Allowing relaxation of the domain wall in the context of the GLD model or even a domain wall topological model [68, 69] may reveal the source of the observed long-range elastic fields.

Finally, it should be emphasized that the results presented here do not address MLCC structures directly, but they do provide a measurement tool and demonstration measurements with the appropriate spatial and deformation resolutions to investigate reliability-limiting features that might arise in commercially fabricated MLCCs. Such MLCCs often use the doped BaTiO_3_ X7R formulation [1, 2], with microstructures significantly affected by the metal electrodes, screen printing of layers, and initial particle size. All these features could influence the formation and motion of domain boundaries and consequent internal stress states in a real MLCC, well beyond the range of structures observed in a single crystal. The work here provides a basis for using EBSD to investigate real MLCC structures.

## 5 Conclusions

Measurements of deformation and stress using high-resolution EBSD can be extended to spatial resolutions of 30 nm with strain and rotation resolutions of approximately 10^−4^ and conjugate stress resolutions of approximately 10 MPa. The capability was demonstrated here on domain features in tetragonal BaTiO_3_. In particular, EBSPs from isolated, sub-micrometer *a* domains, surrounded by 90° domain boundaries within a *c*-domain field, were indexed for orientation, and detailed 2-D maps of strain, rotation, and stress were generated. The maps indicated that the surface deformation of such isolated domains is dominated by out-of-plane rotation, although the in-plane normal strains are still significant. The strain profiles across isolated domains were very similar to those observed across quasi-periodic lamellar and bundled domains, suggesting a commonality in domain boundary structure and within-domain relaxation for different domain microstructures. Maps of principal stress values were generated, indicating tension in the *c*-domain region separating isolated *a* domains. Calculated stress trajectories and assessment of stress values suggested that isolated microcracks in such locations were possible in these BaTiO_3_ microstructures. The surface rotation profile across an isolated *a* domain within a continuous *c*-domain field constrained the subsurface surface termination of the *a* domain to include a dislocation with Burgers vector inclined to the surface. The results clearly demonstrate the applicability of EBSD measurements to commercial MLCC structures and reinforce the linkage between electrical performance of MLCCs, and other BaTiO_3_ devices, and the underlying mechanical features through the domain structure.

## 6 Appendix

This Appendix provides strain information for isolated *a* and *c* domains in barium titanate, supporting claims made in the text. Specifically, Fig. 1A shows strain cross sections taken from Fig. 4 in the text, illustrating the similarity of strain variations within an isolated *a* domain as compared with those previously observed in lamellar and bundled domains and highlighting the resolved strain variations in 30 nm steps. Fig. A2 shows strain maps of an area within Fig. 1(b) of the text, illustrating the lack of strain variation within, and adjacent to, an isolated *c* domain.
